# An in vitro model and the underlying pathways of sinonasal inverted papilloma development

**DOI:** 10.1038/s41598-023-45585-3

**Published:** 2023-10-27

**Authors:** Thawaree Nukpook, Tohru Kiyono, Tipaya Ekalaksananan, Pornthep Kasemsiri, Watchareporn Teeramatwanich, Patravoot Vatanasapt, Surachat Chaiwiriyakul, Tomomi Nakahara, Chamsai Pientong

**Affiliations:** 1 Department of Microbiology, Faculty of Medicine, Khon Kaen University, Khon Kaen, Thailand; 2 HPV & EBV and Carcinogenesis Research Group, Khon Kaen University, Khon Kaen, Thailand; 3 Project for Prevention of HPV-Related Cancer, Exploratory Oncology Research and Clinical Trial Center, National Cancer Center, 6-5-1 Kashiwanoha, Kashiwa, Chiba 277-8577 Japan; 4 Department of Otorhinolaryngology, Faculty of Medicine, Khon Kaen University, Khon Kaen, Thailand; 5 Department of Pathology, Faculty of Medicine, Khon Kaen University, Khon Kaen, Thailand; 6 Division of Immune Medicine, National Cancer Center Research Institute, 5-1-1 Tsukiji, Chuoku, Tokyo 104-0045 Japan

**Keywords:** Experimental models of disease, Cancer models

## Abstract

Recently, the specific association between Sinonasal inverted papilloma (SIP) and EGFR exon 20 mutations has been reported. To investigate the link between specific EGFR mutations and SIP development, we established organotypic raft culture system using nasal polyp-derived immortalized NP2 (iNP2) cells expressing EGFR exon 20 mutants or an exon 19 mutant, and SIP-derived iIP4 cells harboring P772_H773insPYNP mutation. In the raft culture, iIP4 cells showed the inverted growth pattern characteristic to SIP. Interestingly, iNP2 cells expressing EGFR exon 20 duplication mutants, S768_D770dup and N771_H773dup, but not of EGFR exon 19 mutant, E746_A750del, showed the inverted growth pattern. Enhanced activation of the PI3K/AKT signaling pathway was observed in iNP2_S768_D770dup and iIP4 cells, while increased MAPK signaling was found in iNP2_N771_H773dup. Increased cell migration and invasion were found in all cells carrying EGFR mutations when compared to iNP2 cells, and this effect was inhibited by either PI3K or MEK inhibitor. Notably, iNP2 cells expressing the N771_H773dup mutant showed the highest migration and invasion abilities. These results suggest that specific mutations in EGFR exon 20 play a crucial role in SIP development, partially though hyper-activation of the PI3K/AKT and MAPK signaling pathways. This study presents the first in vitro model for SIP development, which could facilitate further investigations into SIP pathogenesis and preclinical studies for new therapeutic agents.

## Introduction

Sinonasal inverted papilloma (SIP) is the most common type of papilloma found in the nasal cavity and paranasal sinus^1^. SIP is a locally aggressive benign tumor with a high recurrence rate (34.09%) and has the potential for malignant transformation^1,2^. The etiology and underlying mechanism of SIP are still under debate^3^. HPV infections have been found to be associated with SIP and may play a role in its pathogenesis^4^. Genetic mutation analysis has revealed the presence of mutations in several genes in SIP, particularly in KRAS, APC and STK11 genes, with increased numbers of genetic mutations seen in SIP-associated squamous cell carcinoma (SCC)^5^. Frequent single nucleotide polymorphisms of TP53, PIK3CA and KRAS genes are observed in SIP and SIP-associated SCC, while insertion-deletion mutations are mainly found in FGFR2 and EGFR genes^5–7^.

Epithelial growth factor receptor (EGFR/ERBB1) is a transmembrane protein belonging to the ERBB family of receptor tyrosine kinases. Activating somatic mutations of EGFR are associated with several tumors^8^. EGFR alterations in tumors are typically linked to a more aggressive phenotype and poor prognosis due to enhanced tumor growth, invasion, and metastasis^9^, making it a key factor in epithelial malignancies.

In SIP, EGFR mutations were mostly insertion in exon 20 with frequencies up to 88% in SIP and 77% in SIP-associated SCC^6,7^. In contrast, non-small cell lung cancer (NSCLC) mostly features EGFR exon 19 deletions, with exon 20 insertions accounting for 4% to 9%. These insertions lead to constitutive activation of EGFR in lung adenocarcinoma^10^. Notably, ERBB1_S768_D770dup is the most common mutation in SIP, while ERBB1_N771_H773dup is frequently found in SIP-associated SCC^7^. These mutations predominantly occur between A767 and C775, which affecting EGFR activation leading to ligand-independent activation^11^. These observations suggest a connection between EGFR exon 20 insertion mutations and SIP pathogenesis. However, the precise roles of EGFR exon 20 mutations in SIP development remain to be elucidated. Therefore, we aimed to establish an in vitro model and unravel the mechanism underlying SIP development, using an organotypic raft culture system to confirm the involvement of specific EGFR mutations in SIP pathogenesis.

## Results

### EGFR exon 20 mutations induced SIP-like growth pattern in iNP2 cell line

**Figure 1:**
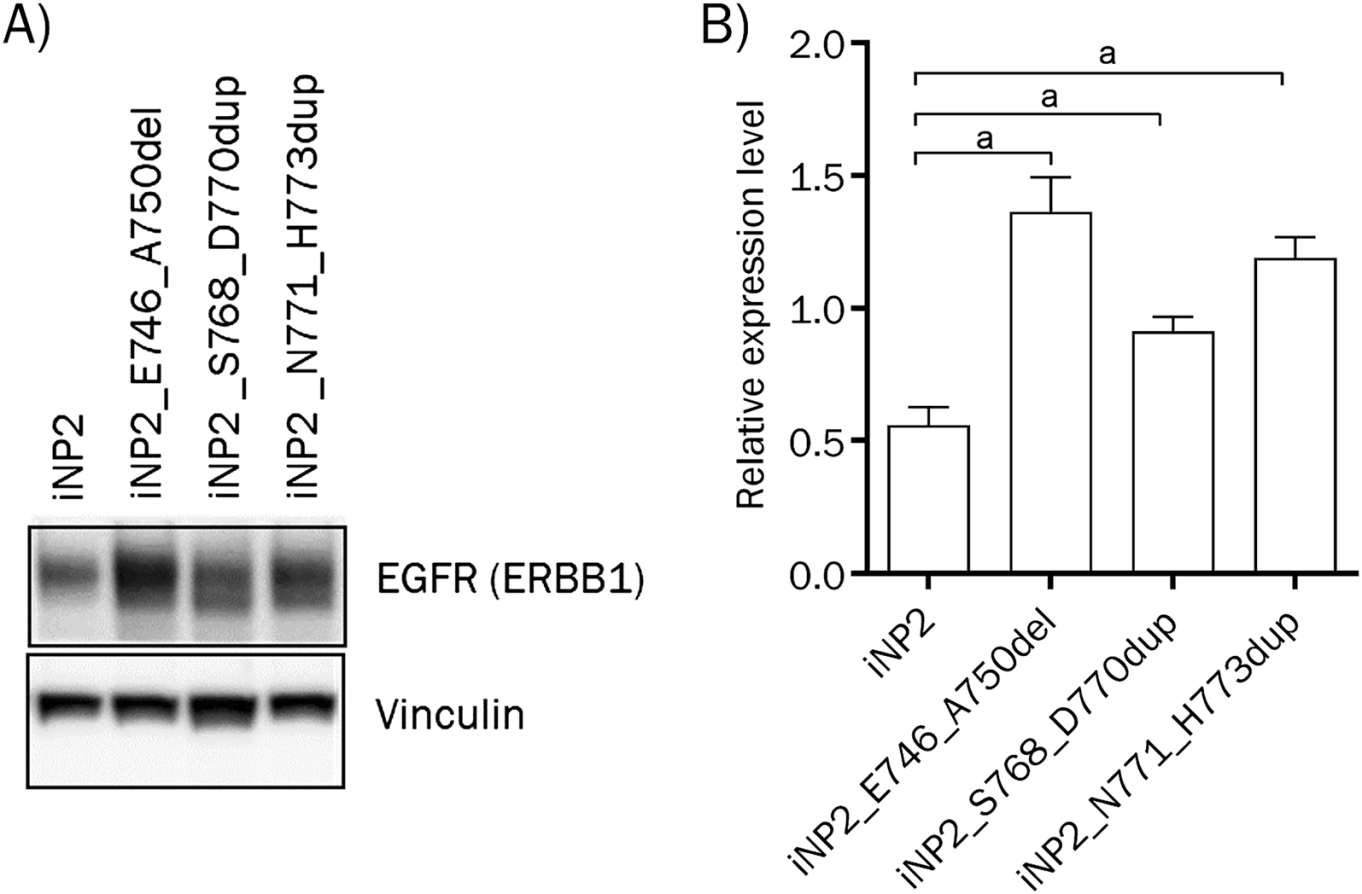
Transgene expression level in transduced iNP2 cells. (**A**) Expression levels of EGFR as well as vinculin as a loading control in iNP2 cells and those transduced with mutant EGFR were examined in triplicate by western blotting. To expose one blot to several antibodies at the same time, the membrane was cut into several pieces at desired positions prior to exposure to specific antibodies. The grouping of blots in (**A**) was cropped from the same samples loaded in 1 gel. The original western blot images of all experimental replicates were presented in the supplementary Fig. S1. (**B**) Band intensities of EGFR (normalized to those of vinculin) were quantified and indicated as bar graphs. “a” indicate statistically significant differences (a: p < 0.05).

**Figure 2:**
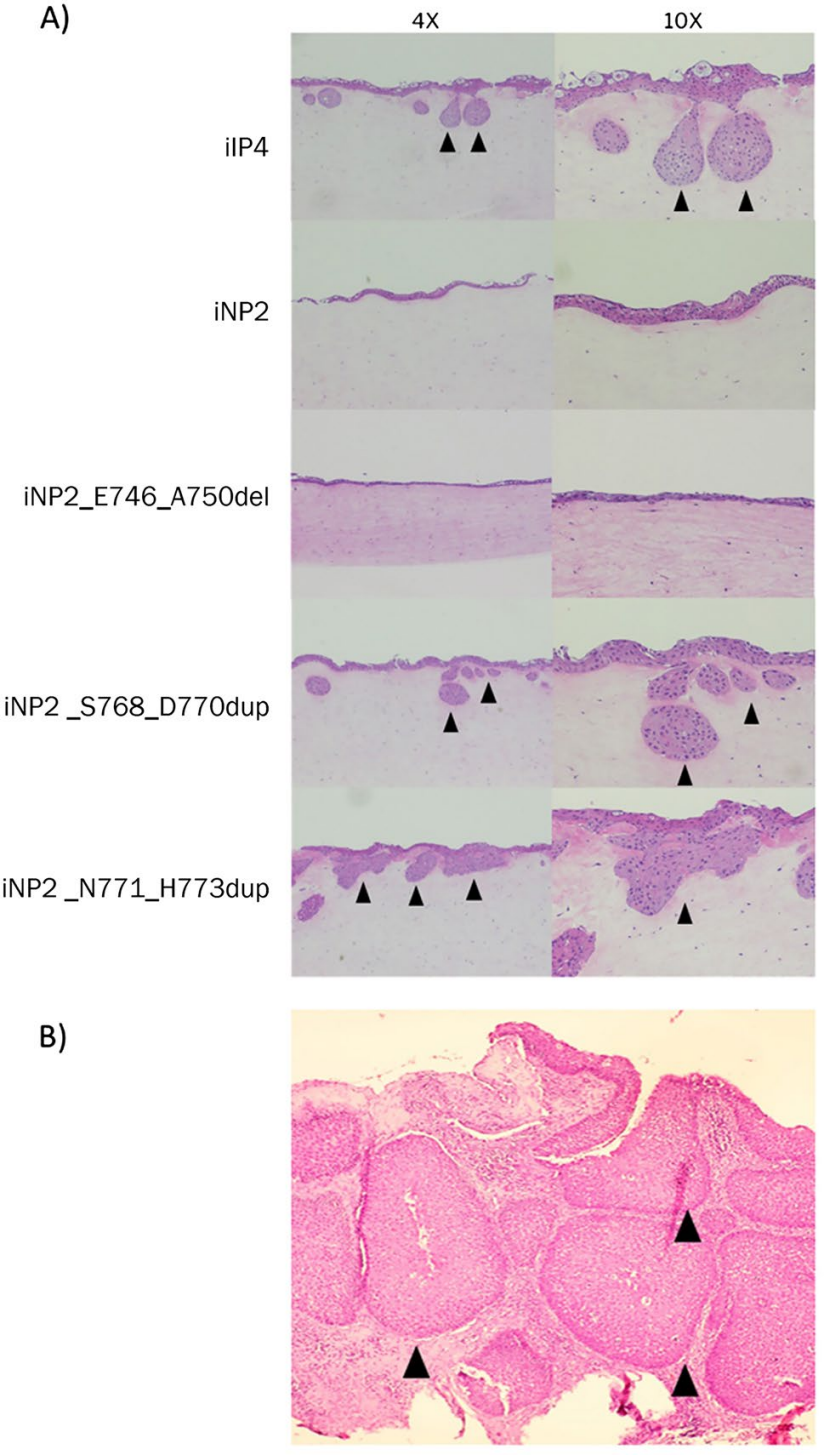
EGFR (ERBB1) exon 20 mutation induces inverted papilloma formation. (**A**) The effect of a specific EGFR exon 20 mutation on sinonasal inverted papilloma formation was investigated by organotypic raft culture. (**B**) H&E staining of a primary tumor where iIP4 cell are derived. Black arrows indicated an inverted growth pattern observed in the raft culture.

To examine the impact of EGFR exon 20 insertion mutations on SIP development, we transduced immortalized nasal polyp cell line, iNP2, with recombinant retroviral vectors carrying EGFR exon 20 insertion mutants, ERBB1_S768_D770dup or ERBB1_N771_H773dup, as well as a typical exon 19 deletion mutant, ERBB1_E746_A750del to generate iNP2_S768_D770dup, iNP2_N771_H773dup, and iNP2_E746_A750del cells. Following puromycin selection, transgene expression levels were confirmed by western blot analysis. Transduced cells displayed higher EGFR expression compared to parental iNP2 cells (Fig. 1, Fig. S1). These cells were subjected to organotypic raft culture to assess SIP-like epithelial growth patterns. Notably, iNP2_S768_D770dup and iNP2_N771_H773dup cells exhibited the inverted growth characteristic resembling SIP, while iNP2_E746_A750del and parental iNP2 cells did not (Fig. 2). As expected, iIP4 cells harboring endogenous EGFR exon 20 insertion mutant^12^, exhibited invasive growth and SIP features (Fig. 2) similar to those seen in iNP2_S768_D770dup and iNP2_N771_H773dup cells in organotypic raft culture. These results suggest that the presence of EGFR exon 20 insertion mutations, rather than exon 19 deletion mutations, is associated with histological characteristics of SIP and may play a significant role in SIP development. However, the underlying molecular mechanisms driving SIP development by EGFR exon 20 mutations need further investigation. Our findings suggest that the organotypic raft culture system holds potential for constructing an in vitro model for SIP development.

### Increased expression of PI3K, p-AKT, and p-MAPK induced by EGFR exon 20 mutations associated with SIP development

**Figure 3:**
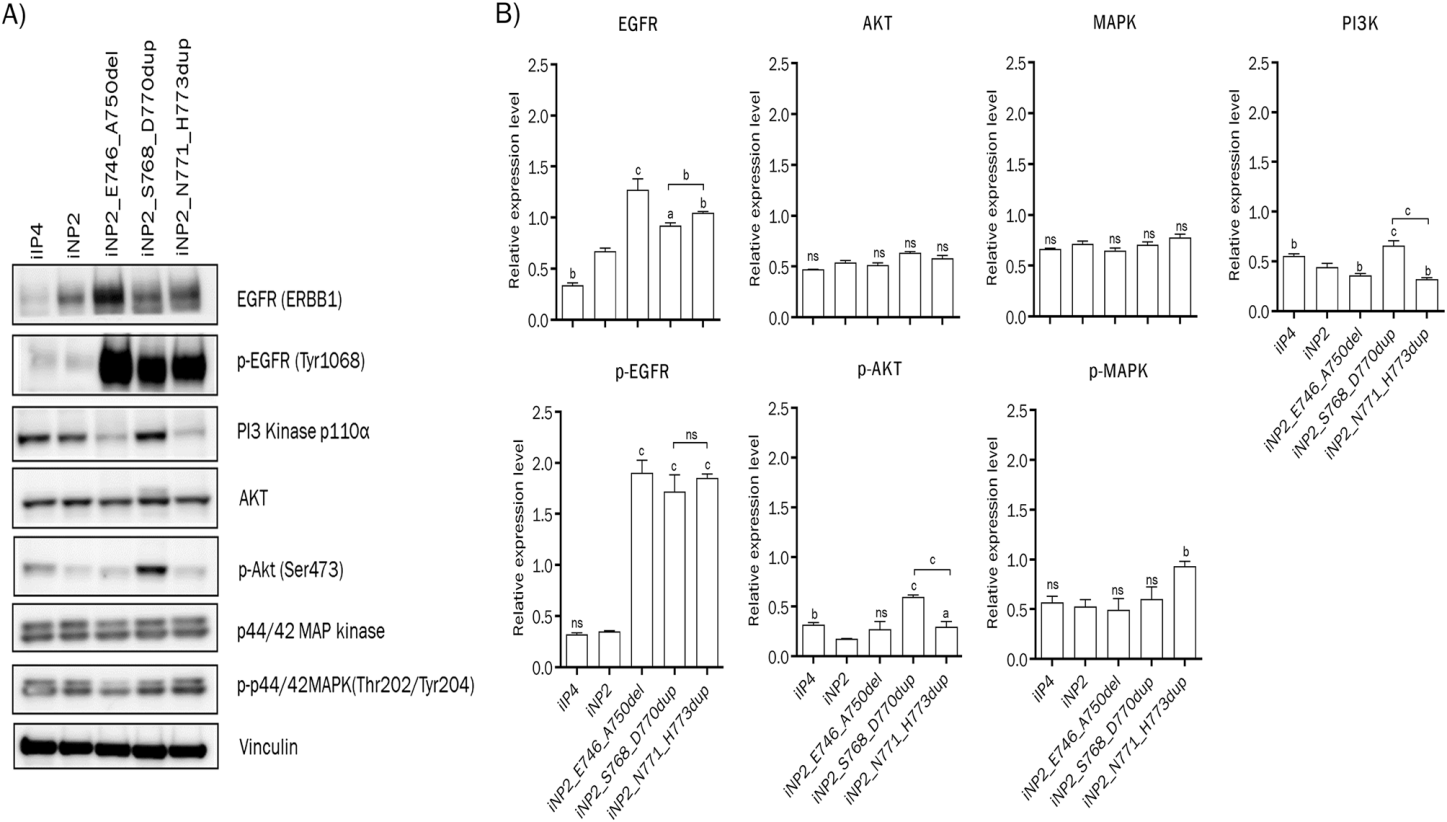
Activation of PI3K/AKT signaling and MAPK signaling pathway in SIP. (**A**) The expression of total and phosphorylated proteins of EGFR, PI3K, AKT, and MAPK were investigated in iIP4, iNP2 and iNP2 cell carrying EGFR (ERBB1) mutation using western blotting as described in Methods. To expose one blot to several antibodies at the same time, the membrane was cut into several pieces at desired positions prior to exposure to specific antibodies. The grouping of blots in (**A**) was cropped from the same samples loaded in 2 gels with 8 blots. The original western blot images of all experimental replicates were presented in the supplementary Fig. S2. (**B**) The intensities of individual bands were indicated by bar graphs. “a, b, c” indicate a statistically significant difference (a: p < 0.05; b: p < 0.01; c: p < 0.001), and “ns” indicates not significant.

To investigate the possible mechanism by which EGFR exon 20 mutations induce SIP development, we compared the expression levels of downstream signaling proteins of EGFR in iNP2, iNP2_S768_D770dup, iNP2_N771_H773dup, iNP2_E746_A750del, and iIP4 cells. Increased levels of PI3K and p-AKT were observed in iNP2_S768_D770dup and iIP4 cells, while increased p-AKT and p-MAPK were detected in iNP2_N771_H773dup cells that showed the inverted growth pattern in organotypic raft culture, compared to iNP2 cells (Fig. 3, Fig. S2). These results suggest that hyper-activation of PI3K/AKT and MAPK signaling pathways, driven by EGFR exon 20 insertion mutations may contribute to SIP development.

### EGFR exon 20 mutations increased the invasiveness of SIP

**Figure 4:**
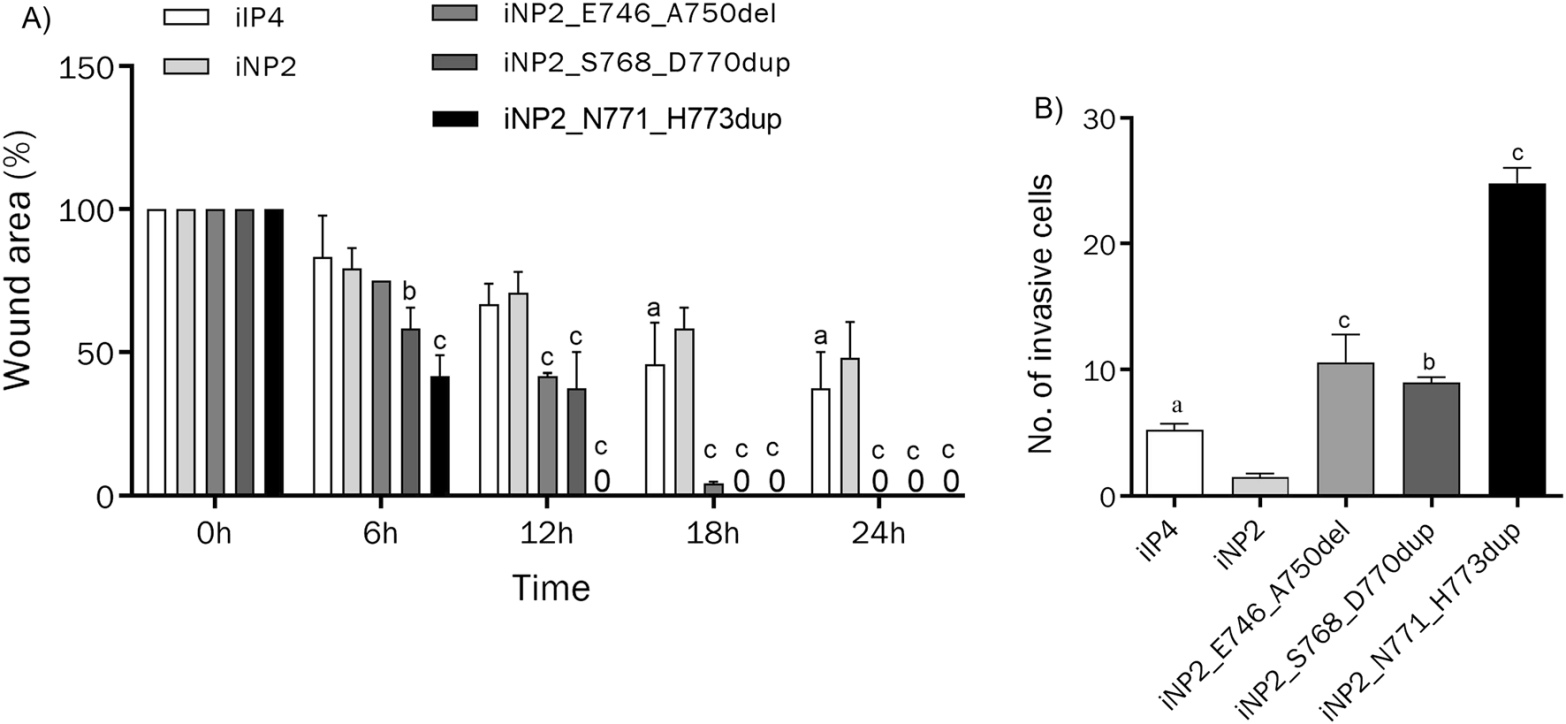
Migration and invasion abilities of inverted papilloma cells. (**A**) Migration and (**B**) invasion abilities were measured by wound healing and transwell invasion assay, respectively, as described in “Methods”. Note that iNP2_N771_H773dup showed greater migration and invasion abilities compared to other cells. “a, b, c” indicate a statistically significant difference (a: p < 0.05; b: p < 0.01; c: p < 0.001).

As the growth pattern of SIP topologically bears resemblance to cancer invasion, we investigated the effect of EGFR exon 20 mutations on cell migration and invasion abilities of cells exhibiting SIP-like growth pattern. We performed wound healing and transwell invasion assays. All EGFR exon 20 mutant cells displayed enhanced migration and invasion abilities compared to wild-type EGFR iNP2 cells (Fig. 4). Notably, similar results were observed in EGFR exon 19 mutant cells, despite the absence of the inverted growth pattern characteristic of SIP in organotypic raft culture. Interestingly, iNP2_N771_H773dup, which exhibited more aggressive inverted growth features, displayed the highest cell migration and invasion abilities. These findings underscore the specific role of EGFR exon 20 insertion mutations in promoting invasiveness and development of SIP. The greater effect on triggering invasive property might be the reason why SIP patients who carry ERBB1_N771_H773dup have a higher progression rate from SIP to SIP-associated SCC.

### Inhibition of the PI3K/AKT and MAPK signaling pathways reduced cell migration and invasion

**Figure 5:**
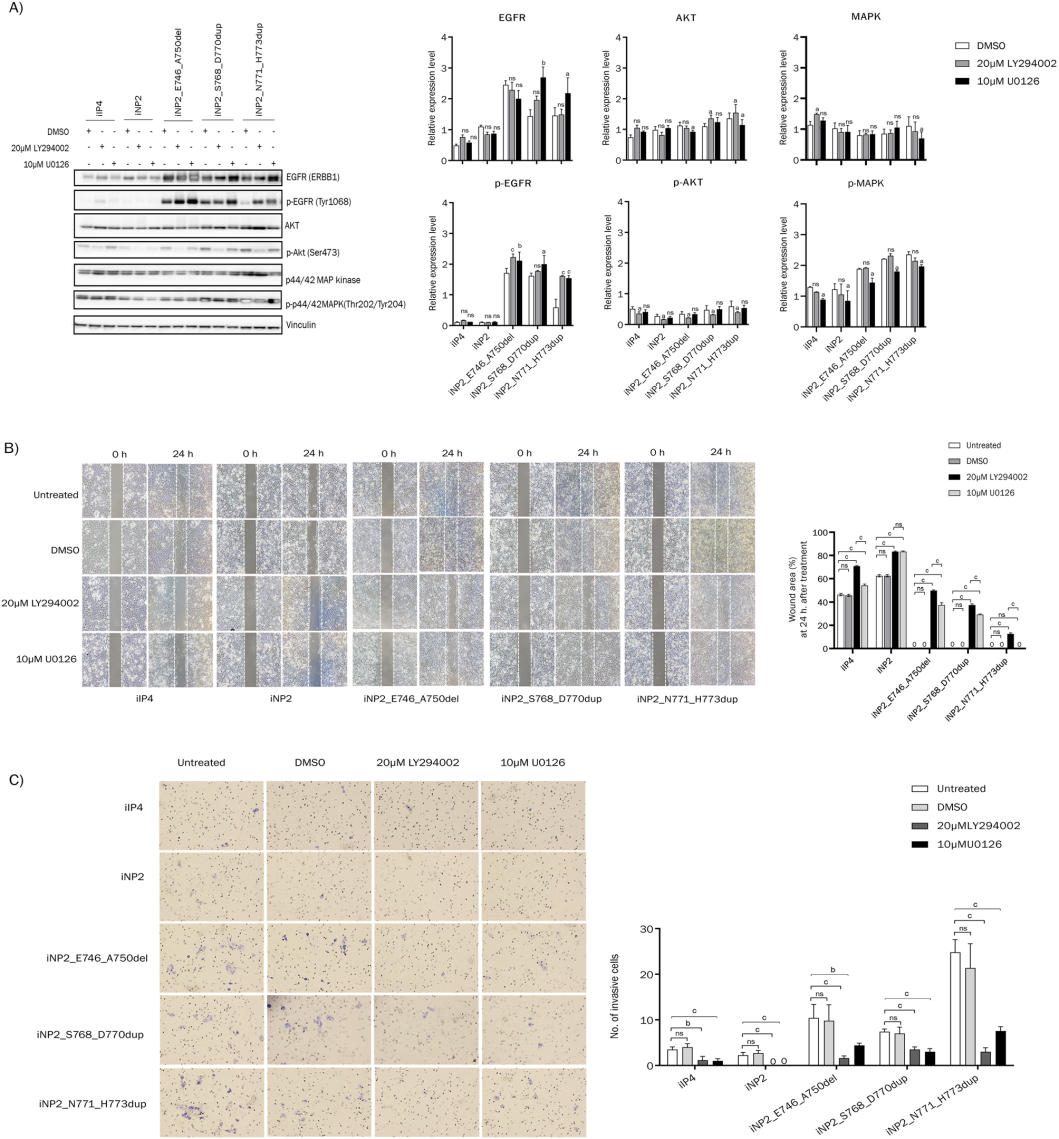
A PI3K inhibitor and MEK inhibitor inhibited migration and invasion abilities of SIP cell, iNP cell and iNP cell carrying EGFR (ERBB1) mutation. (**A**) The cells were cultured in 6-well plate, starved overnight in serum- and EGF-free FYAD medium, then incubated in the presence or absence of 20 µM LY294002 or 10µM U0126 for 48 h prior to harvesting cell lysates and analyzed by western blotting. The total and the phosphorylated levels of EGFR, AKT, and MAPK were detected by the antibodies described in “Methods” (left panel), and the intensities of individual bands were indicated by bar graphs (right panels). To expose one blot to several antibodies at the same time, the membrane was cut into several pieces at desired positions prior to exposure to specific antibodies. The grouping of blots in (**A**) was cropped from the same samples loaded in 2 gels with 7 blots. The original blots are presented in Supplementary Fig. S3. (**B**) Cell migration was investigated by wound healing assay as described in “Method” (left panel), and the wound area at 24 h after 20 µM LY294002 or 10 µM U0126 treatment were indicated by bar graphs (right panels). (**C**) Cell invasion was investigated by using transwell invasion assay as described in “Method” (left panel), and the number of invaded cells at 24 h after 20 µM LY294002 or 10 µM U0126 treatment were indicated by bar graphs (right panels). Note that treatment with 20 µM LY294002 and 10 µM U0126 significantly reduced migration and invasion abilities of the tested cells. “a, b, c” indicate a statistically significant difference (a: p < 0.05; b: p < 0.01; c: p < 0.001), and “ns” indicates not significant.

It is well known that alteration of PI3K/AKT and MAPK signaling pathways are associated with cell migration and invasion abilities^13,14^. Since both EGFR exon 19 and EGFR exon 20 mutant cells showed increased migration and invasion compared to iNP2 cells (Fig. 4), and upregulation of PI3K, p-AKT, and p-MAPK was observed in cells exhibiting inverted growth patterns in organotypic raft culture (Fig. 3), we explored the role of the PI3K/AKT and MAPK signaling pathways in SIP cells’ migration and invasion. We treated iIP4, iNP2, iNP2_S768_D770dup, and iNP2_N771_H773dup cells as well as iNP2_E746_A750del cells, which did not display the inverted growth pattern, with the PI3K inhibitor LY294002 or the MEK inhibitor U0126. Decreased levels of p-AKT and p-MAPK after treatment with LY294002 or U0126 in all tested cells were confirmed by western blot analysis (Fig. 5A, Fig. S3). Wound healing results demonstrated inhibited cell migration in the presence of LY294002 or U0126 compared to the vehicle (Fig. 5B). Similarly, the transwell invasion assay revealed reduced invasion rates in cells treated with LY294002 or U0126 compared to the vehicle (Fig. 5C). These results indicated that increased PI3K/AKT and MAPK signaling activation due to EGFR mutations is associated with cell migration and invasion abilities which can be suppressed by PI3K and MEK inhibitors.

## Discussion

In this study, we investigated the involvement of EGFR exon 20 mutations in initiating SIP development using an organotypic raft culture system. Intriguingly, an inverted growth pattern was observed in iNP2 cells carrying EGFR exon 20 mutants (ERBB1_S768_D770dup and ERBB1_N771_H773dup) and iIP4 cells, but not in parental iNP2 cells or EGFR exon 19 mutant (ERBB1_E746_A750del) cells (Fig. 2). This result is in line with the previous reports that duplication/insertion mutations of EGFR exon 20 are specifically associated with SIP^7,15^. Our results emphasize the significant role of EGFR exon 20 insertion mutations in SIP development, as demonstrated by this in vitro model.

Alteration of EGFR and its downstream signaling activation disrupts various cellular processes, contributing to tumorigenesis in several cancers^14,16–18^. We observed higher expression levels of EGFR and pEGFR in all mutant EGFR-transduced iNP2 cells compared to iIP4 cells (Fig. 3). The difference probably arises from the stronger CMV promoter driving mutant EGFR expression, as opposed to the endogenous EGFR promoter used in iIP4 cells. These findings align with a previous study reporting low to moderate EGFR expression in SIP tissues^19^. The difference in promoter activities likely led to a difference in migration and invasion abilities between iIP4 cells and iNP2 cells expressed the same EGFR exon 20 mutant (Fig. 2). None the less, they exhibited similar histological inverted patterns. Hyperactivation of PI3K/AKT and MAPK signaling caused by EGFR mutations can stimulate proliferation, invasion, and migration abilities of cancer cells^20–23^, with these effects being suppressed by PI3K and MEK inhibitors^24^. SIP tissues display increased cell proliferation compared to nasal polyps^25^. In HPV-associated SIP, HPV promotes cell proliferation via the AKT/mTOR signaling pathway^26^. Hyperactivated EGFR can trigger EGFR negative feedback loops^27^, i.e. an EGFR downstream target, ERRFI1, acts as an EGFR inhibitor in EGFR-high-expressing cells, while activating AKT signaling in EGFR-low-expressing cells^28^. This information might account for the slight difference in pAKT and pMAPK expressions observed in all mutant EGFR-transduced iNP2 cells compared to iIP4 and iNP2 cells (Fig. 3). Interestingly, increased AKT phosphorylation is observed in SIP and SIP-associated SCC with EGFR exon 20 mutations, regardless of EGFR expression levels^29^. Similarly, high levels of PI3K, p-AKT, and p-MAPK were observed in all cells displaying inverted growth compared to iNP2 cells (Fig. 3). Increased cell migration and invasion were evident in these cells (Fig. 4), which were inhibited by PI3K and MEK inhibitors (Fig. 5). These lines of evidence suggest that hyperactivation of PI3K/AKT and MAPK signaling might serve as a switch regulating cell proliferation, invasion, and migration in SIP. Our findings suggest a connection between hyperactivation of PI3K/AKT and MAPK signaling and SIP development, with EGFR exon 20 insertion mutations promoting this activation.

SIP is considered a precursor for SNSCC^30^. EGFR exon 20 mutations, particularly ERBB1_N771_H773dup, are suggested as early events in SIP malignant transformation^7^. This is in line with our finding that iNP2_N771_H773dup displayed a more aggressive phenotype compared to iNP2_S768_D770dup and iIP4. Recently it was reported that PIK3CA mutations, EGFR copy number gains, and at least one TP53 or CDKN2A alteration were commonly observed^31^, supporting the importance of hyperactivation of PI3K/AKT in addition to inactivation of p53 or p16 in malignant conversion of SIP. In this regard, iNP2_N771_H773dup cells might be more like SIP-associated SCC as the p16/pRb pathway was inactivated by CDK4R24C and cyclin D1, which had been transduced for immortalization, and the expression level of ERBB1_N771_H773dup was much higher than that of iNP2_S768_D770dup, and iIP4, which mimicked copy number gain of the mutant ERBB1. However, additional events such as TP53 mutations or CDKN2A alterations appear essential for SIP-associated SCC malignant transformation^31^. SIP-associated SCC and papilloma exhibit distinct histological features, typically showing an abrupt transition between the two components^32^.

While iNP2 and iIP4 cells were immortalized with three transgenes, CDK4R24C, cyclin D1 and TERT to overcome p16INK4a accumulation due to culture stress and telomere shortening, they maintained characteristics of parental cells^12^. iNP2 cells did not display any transformed phenotype, including inverted growth. Therefore, iNP2 cells could serve as a model to study the effect of EGFR exon 20 mutations on SIP development. However, it’s important to note that the transgenes used in the immortalization system can play a role in squamous cell carcinomas. Though EGFR exon 20 mutations are most frequently observed in SIP, other EGFR variants can be detected^7,29^. Comprehensive understanding of mechanisms driving SIP’s inverted growth patterns necessitates investigating the influence of other potential etiological factors and different EGFR mutation variants.

**Figure 6:**
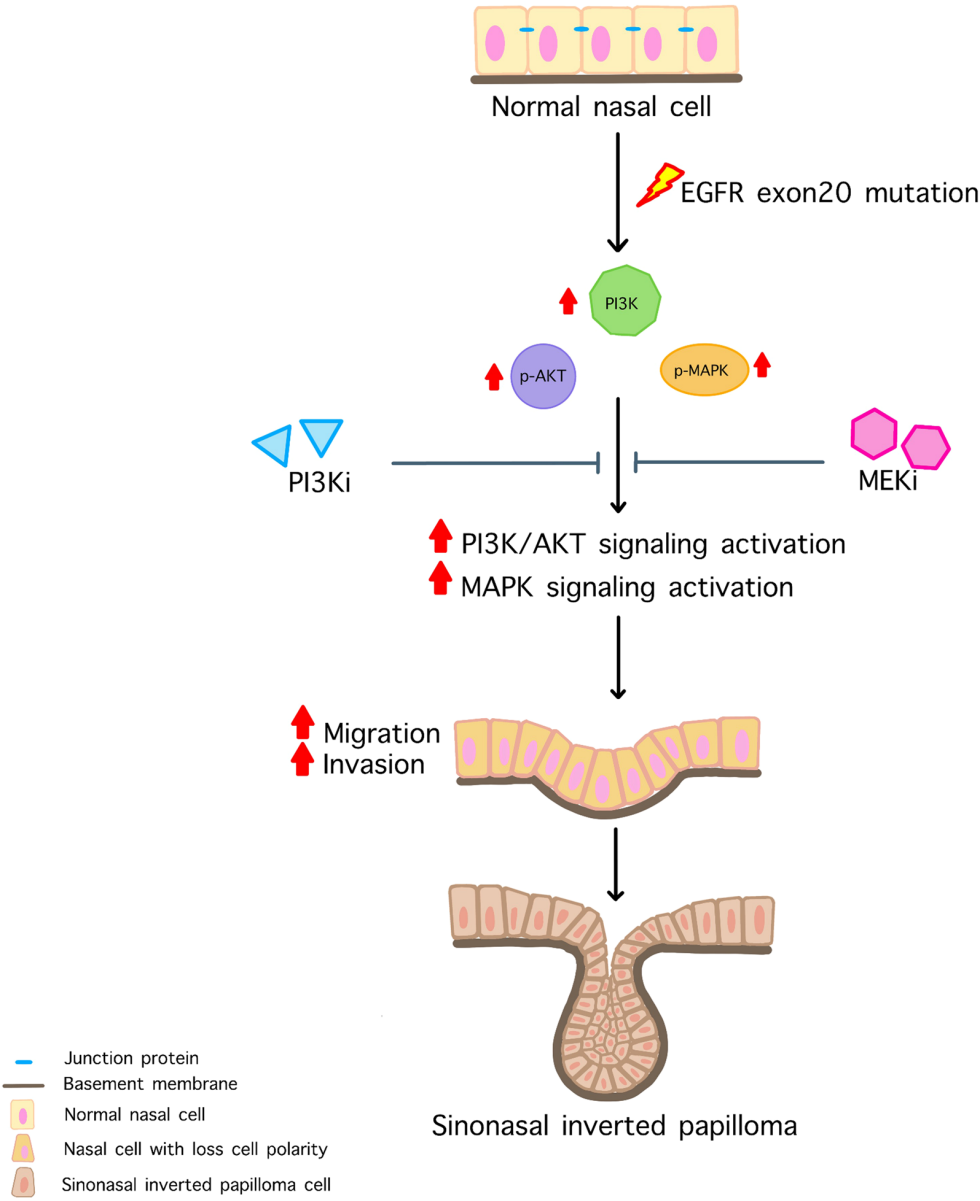
Possible mechanism of EGFR exon 20 mutation induced SIP development. The specific mutation of EGFR in epithelial cells leading to ligand-independent activation of EGFR. Uncontrol EGFR activation resulting in increased activation of PI3K/AKT and MAPK signaling pathways. Aberrant activation of these signaling pathways involves several cellular processes including cell proliferation, migration, and invasion. In SIP, EGFR exon 20 mutations induce increased expression of PI3K, p-AKT, and p-MAPK. After PI3K/AKT and MAPK signaling pathways are activated leading to increased cell migration and invasion abilities of the cells. This aggressiveness might be the factor that involved or enhanced the cells to grow invert into the underlying supportive tissue which is the characteristic of SIP.

In summary, this study highlights the utility of an in vitro model for SIP using an organotypic raft culture system. We underscore the critical role of EGFR exon 20 insertion mutations in the SIP pathogenesis and propose a potential mechanism whereby EGFR exon 20 mutations induce SIP development through PI3K/AKT and MAPK signaling pathways (Fig. 6). To the best of our knowledge, this is the first study establishing a dedicated in vitro model for SIP development.

## Material and methods

### Cell lines

NP2/K4DT, human nasal polyp cell line contained wild type EGFR, and IP4/K4DT, human sinonasal inverted papilloma cell line contained endogenous EGFR exon 20 insertion mutation at ERBB1_P772_H773insPYNP. These immortalized cell lines are obtained by transducing the primary IP4 and NP2 cells with CDK4R24C, cyclin D1 and TERT as previously described^12^. The cells were cultured in FYAD medium [F culture medium^33^ supplemented with 10 µM Y-27632 (Selleck, USA), 500 nM A-83-01 (TOCRIS, UK) and 500 nM DMH-1 (Selleck, USA)], maintained at 37 °C in a humidified atmosphere containing 5% CO_2_. To shorten the cells' names, NP2/N4DT and IP4/K4DT cells were renamed iNP2 and iIP4, respectively.

Normal human foreskin fibroblast cell line, HFF2T was cultured in DMEM supplemented with 10% FBS, maintained at 37 °C in a humidified atmosphere containing 5% CO_2_.

### Viral vector construction and viral transduction

Retroviral expression vectors contained EGFR exon 19 mutation; PQCXIP-ERBB1_E746_A750del^34^, and exon 20 nutation; PQCXIP-ERBB1_S768_D770dup, and PQCXIP-ERBB1_N771_H773dup were constructed. These mutated genes were generated by site-directed mutagenesis using pENTR221-ERBB1 wild type as a template and then recombined with the retroviral vector pDEST-PQCXIP by the LR reaction (Invitrogen, USA). The production of recombinant retroviruses was as described previously^35^. The viral fluids were inoculated to the iNP2 cells in the presence of 4 µg/ml polybrene for 24 h and replaced with fresh media. The infected cells were selected in the presence of 1 μg/ml of puromycin. The generated cells are called iNP2_E746_A750del, iNP2_S768_D770dup, and iNP2_N771_H773dup, respectively. Protocol and primers used to generate EGFR mutants were shown in the supplementary information.

### Organotypic raft culture

Normal human foreskin fibroblast cell line, HFF2T was used to make collagen-fibroblast plugs as described previously^36^ with the final collagen concentration of 2 mg/ml. The keratinocyte cells, iIP4, iNP2, iNP2_E746_A750del, iNP2_S768_D770dup, and iNP2_N771_H773dup were cultured in FYAD medium prior to seeding onto the collagen plugs. After keratinocyte cells were trypsinized, 2 × 10^5^ cells in 200 µl of FYAD medium were seeded inside a glass ring placed on the surface of the collagen plugs in the upper chamber, while lower chamber was filled with 1.5 ml of FYAD medium and maintained at 37 °C in a humidified chamber supplemented with 5% CO_2_. The next day, the glass ring was removed and allowed cells to grow to confluence. The media from the top chamber of the transwell was removed without disturbing the collagen plug or the keratinocyte sheet to create an air–liquid interface. The media in the lower chamber was replaced with 1.5 ml F-media w/o EGF, 1.8 mM Ca^2+^ (differentiation media) to allow cell differentiation. The media was changed every 2 days thereafter (Organotypic culture was obtained by 7 to 14 days).

### Western blot analysis

The expression levels of EGFR, p-EGFR, PI3K, AKT, p-AKT, MAPK, and p-MAPK as well as vinculin were examined by western blotting. The western blot was performed as described previously^37^. To expose one blot to several antibodies at the same time, the membrane was cut into several pieces at desired positions prior to exposure to specific antibodies. Antibodies against EGFR (Cell Signaling Technology, USA, Cat# 2646), p-EGFR (Cell Signaling Technology, USA, Cat# 2234), PI3K (Cell Signaling Technology, USA, Cat# 4249,), AKT (Cell Signaling Technology, USA, Cat# 9272S), p-AKT (Cell Signaling Technology, USA, Cat#9271S), MAPK (Cell Signaling Technology, USA, Cat# 9102,), p-MAPK (Cell Signaling Technology, USA, Cat# 9101) and vinculin (Sigma-Aldrich, USA, Cat# V9264) were used as primary antibodies. Horseradish peroxidase-conjugated anti-mouse or anti-rabbit immunoglobulins (Jackson Immunoresearch Laboratories, West Grove, PA) were used as secondary antibodies.

### Cell migration and invasion assay

iIP4, iNP2, iNP2_E746_A750del, iNP2_S768_D770dup, and iNP2_N771_H773dup cells were cultured in FYAD medium until the cells became nearly confluence, then the culture media was replaced with low serum or growth factor medium for overnight. For cell migration assay, wounds were incised with a sterile 200 µl pipette tip in the central area of culture and the culture media was replaced with 2 ml of FYAD medium/2%FBS/without EGF containing 20 µM LY294002, 10 µM U0126 or vehicle (DMSO). Wound healing was measured every 6 h. until 24 h. For invasion assay, 1 × 10^5^ cells in 200 µl of serum-free medium containing 20 µM LY294002, 10 µM U0126 or vehicle was plated in the upper chamber coated with 50 µl of 1 mg/ml Matrigel matrix, whereas 700 µl of medium/5% fetal bovine serum containing 20 µM LY294002, 10 µM U0126 or vehicle were added to the lower chamber. The cells were incubated for 24 h at 37 °C with 5% CO_2_. The cells that did not penetrate the membrane were removed with a cotton swab, whereas invading cells were fixed with 4% paraformaldehyde for 25 min and stained for 15 min with 1% crystal violet dye. The invaded cells were counted in five or six randomly selected fields per chamber.

### Statistical analysis

Expression differences between parental and their derivatives were calculated using the student’s t-test. Cell migration, invasion, and protein expression in different cell lines were analyzed by ANOVA using STATA. Differences were considered statistically significant at p < 0.05. Data are presented as the mean ± SD.

### Supplementary Information

Supplementary Information.

## Data Availability

The pENTR221-ERBB1 vector sequence is available in figshare (https://doi.org/10.6084/m9.figshare.21988247.v1). All other data analyzed during this study are included in this published article and its supplementary information files.
