# Supplementary material for: An in vitro model and the underlying pathways of sinonasal inverted papilloma development
Source: Sci Rep. 2023 Oct 27;13:18456. doi: 10.1038/s41598-023-45585-3 (PMC10611779; doi:10.1038/s41598-023-45585-3)
Supplement: Supplementary file 1 — Supplementary Information. [file 41598_2023_45585_MOESM1_ESM.pdf]

An *in vitro* model and the underlying pathways of sinonasal inverted papilloma development

Thawaree Nukpook[a,b], Tohru Kiyono[c,*], Tipaya Ekalaksananan[a,b], Pornthep Kasemsiri[d], Watchareporn Teeramatwanich[b,d], Patravoot Vatanasapt[b,d], Surachat Chaiwiriyakul[e], Tomomi Nakahara[f], Chamsai Pientong[a,b,*]

[a] Department of Microbiology, Faculty of Medicine, Khon Kaen University, Khon Kaen, Thailand

[b] HPV & EBV and Carcinogenesis Research Group, Khon Kaen University, Khon Kaen, Thailand

[c] Project for Prevention of HPV-related Cancer, Exploratory Oncology Research and Clinical Trial Center, National Cancer Center, 6-5-1 Kashiwanoha, Kashiwa, Chiba 277-8577, Japan

[d] Department of Otorhinolaryngology, Faculty of Medicine, Khon Kaen University, Khon Kaen, Thailand

[e] Department of Pathology, Faculty of Medicine, Khon Kaen University, Khon Kaen, Thailand

[f] Division of Immune Medicine, National Cancer Center Research Institute, 5-1-1 Tsukiji, Chuoku, Tokyo 104-0045, Japan

*Corresponding author

E-mail: chapie@kku.ac.th; tkiyono@east.ncc.go.jp

Full images of Western blot pictures

Original blots in different times (a time-lapse sequence)

15 Sec

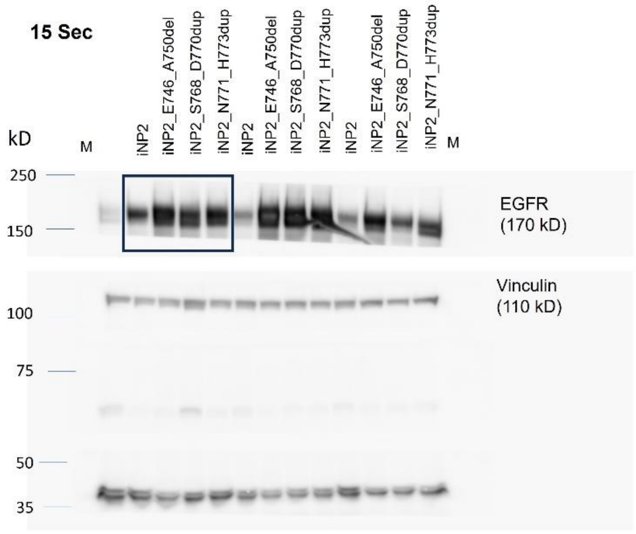

30 Sec

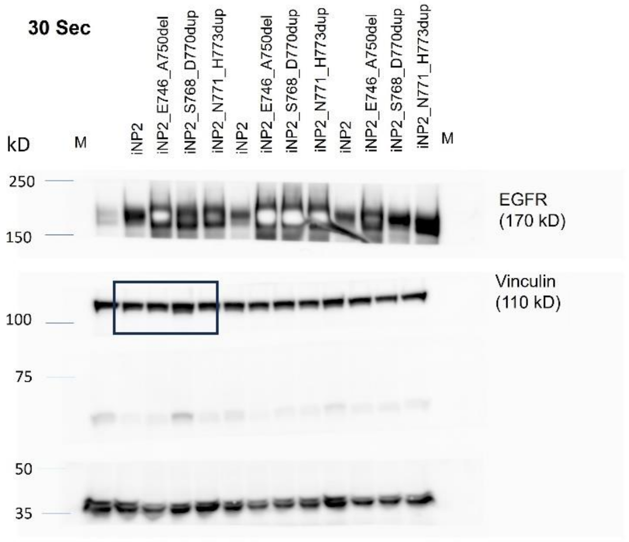

2 Min

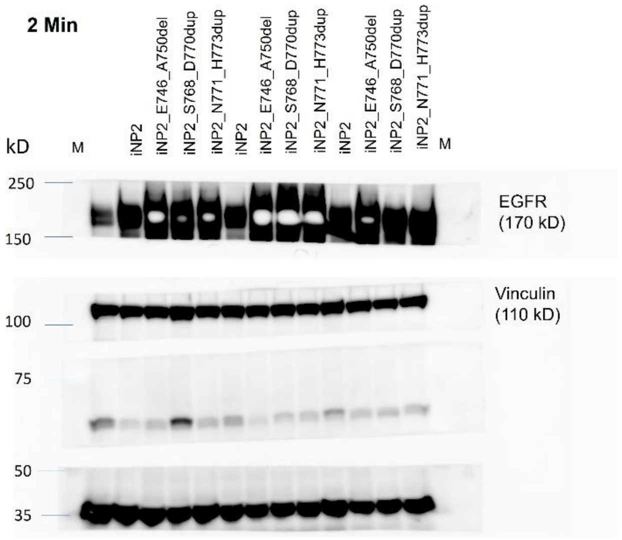

4 Min

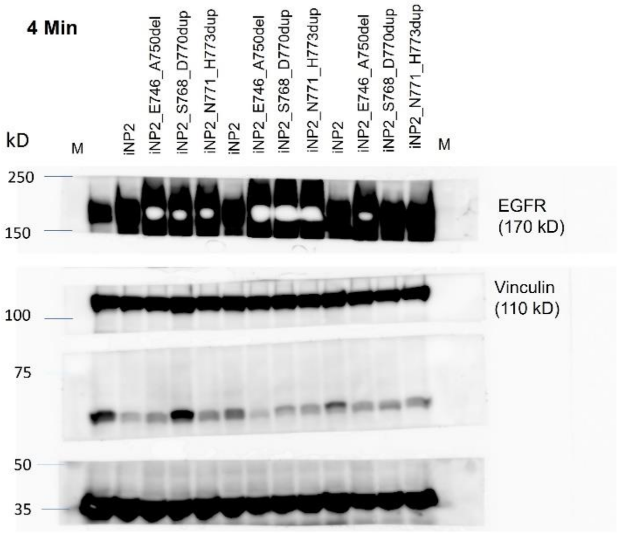

Cropped image in Figure 1A

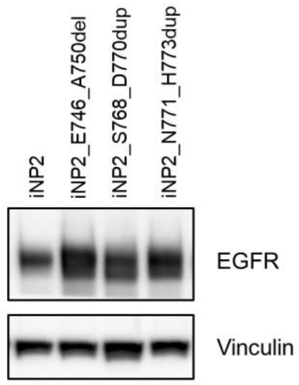

Figure S1. Full images of Western blot in Figure 1A. After the western blot was performed as described in Materials and Methods, to hybridize one blot with several antibodies at the same time, the membrane was cut into several pieces at desired positions prior to hybridization with specific antibodies. The figure shows the original images of western blot in different exposure times (15 sec to 4 minutes), the grouping of blots in Figure 1A was cropped from the same samples loaded in 1 gel. The black box in the original pictures indicated a group of the cropped blots that show in Figure 1A.

Replicate: 1

Original blots in different times (a time-lapse sequence)

250 ms

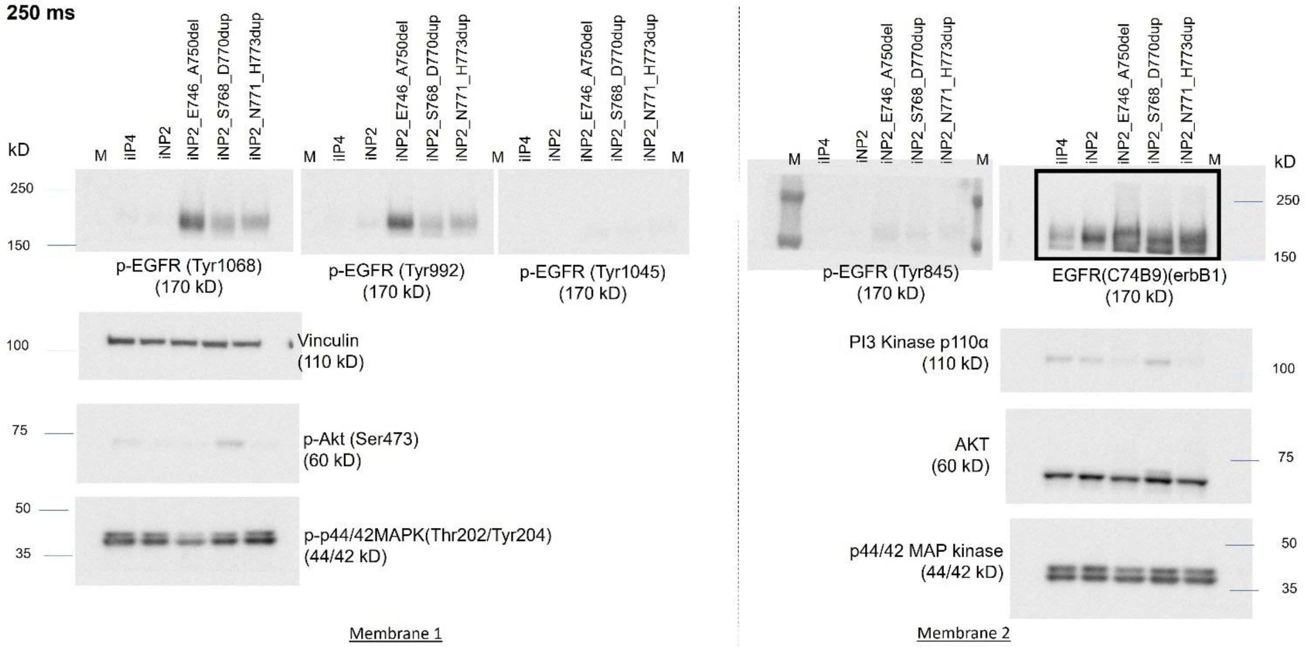

10 Sec

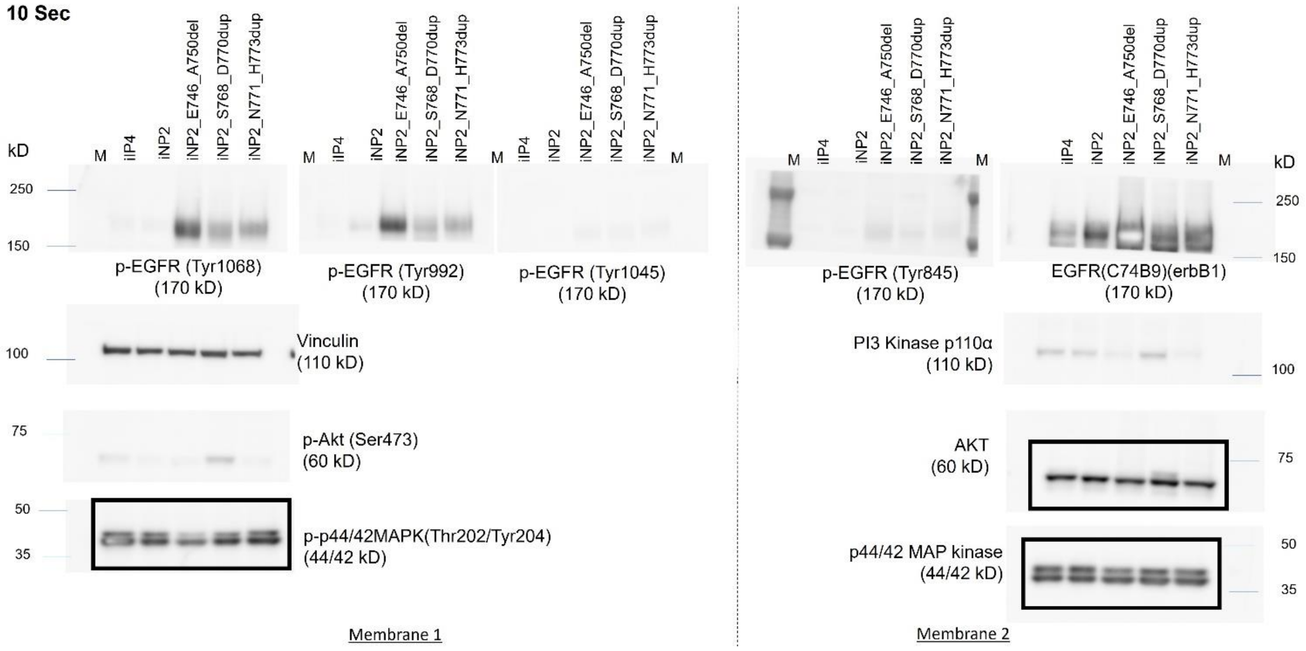

2 Min

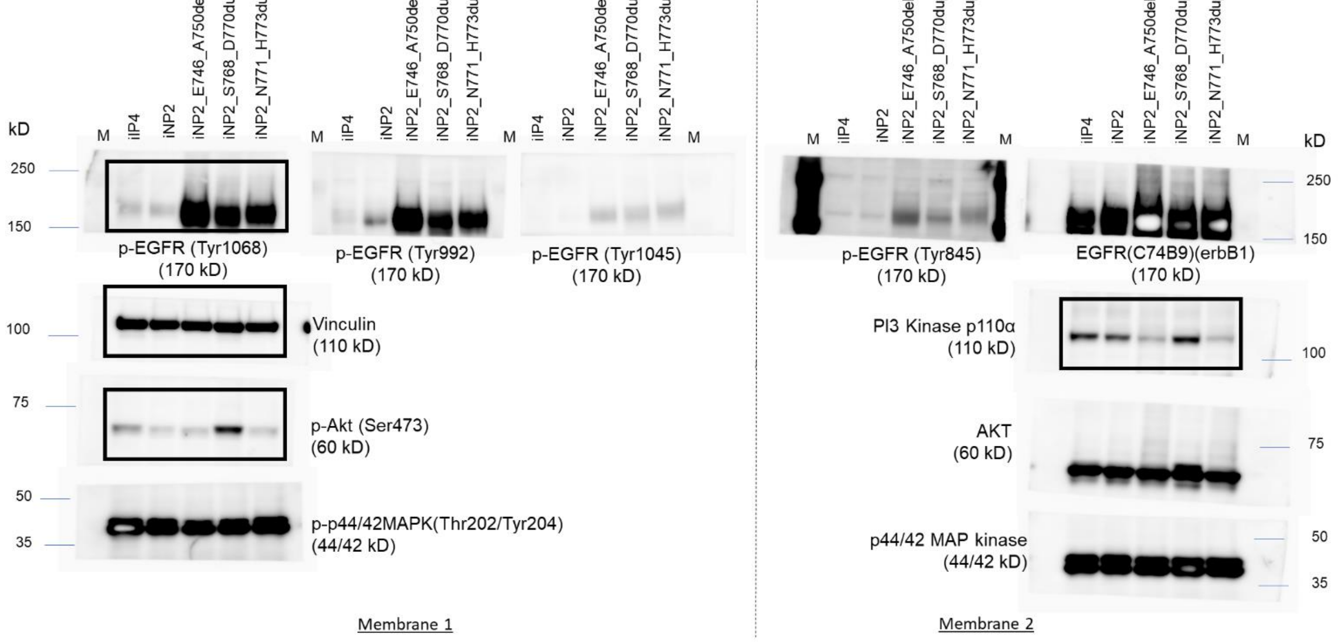

4 Min

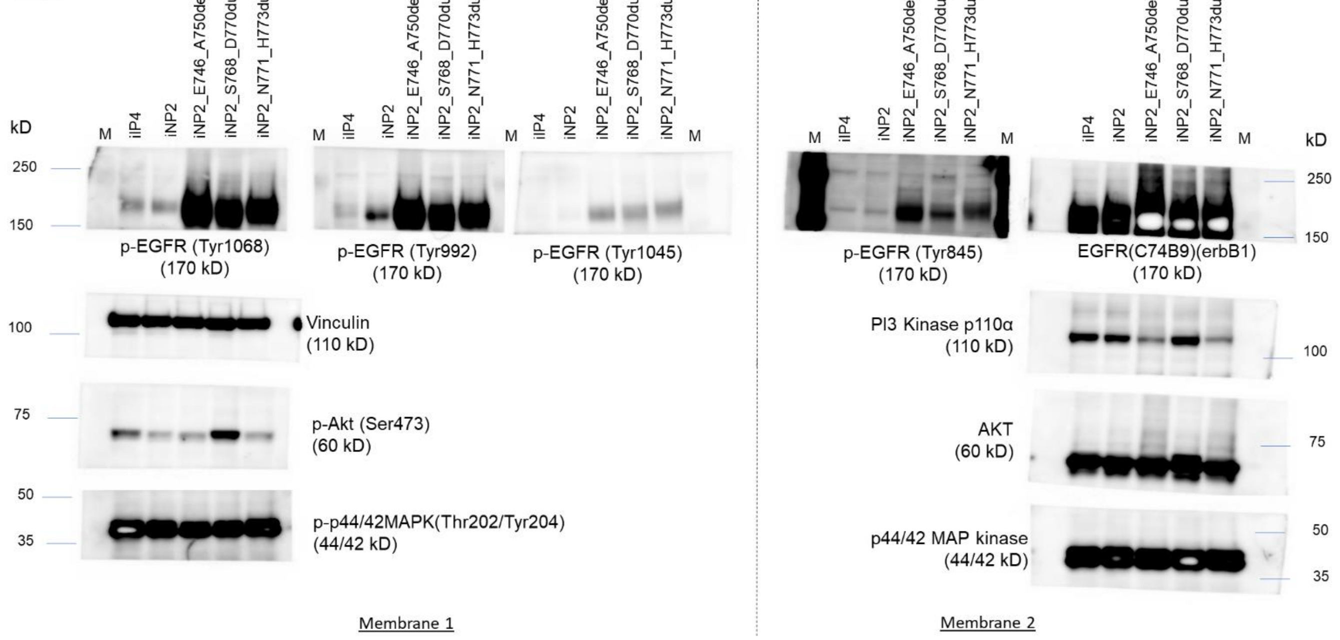

Cropped image in Figure 3A

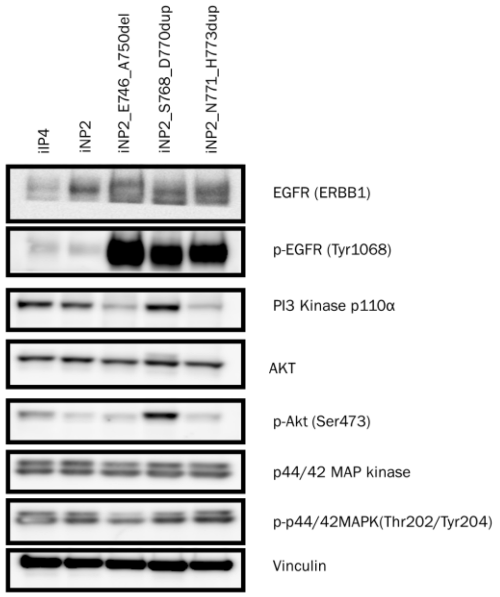

Replicate: 2

Original blots in different times (a time-lapse sequence)

250 ms

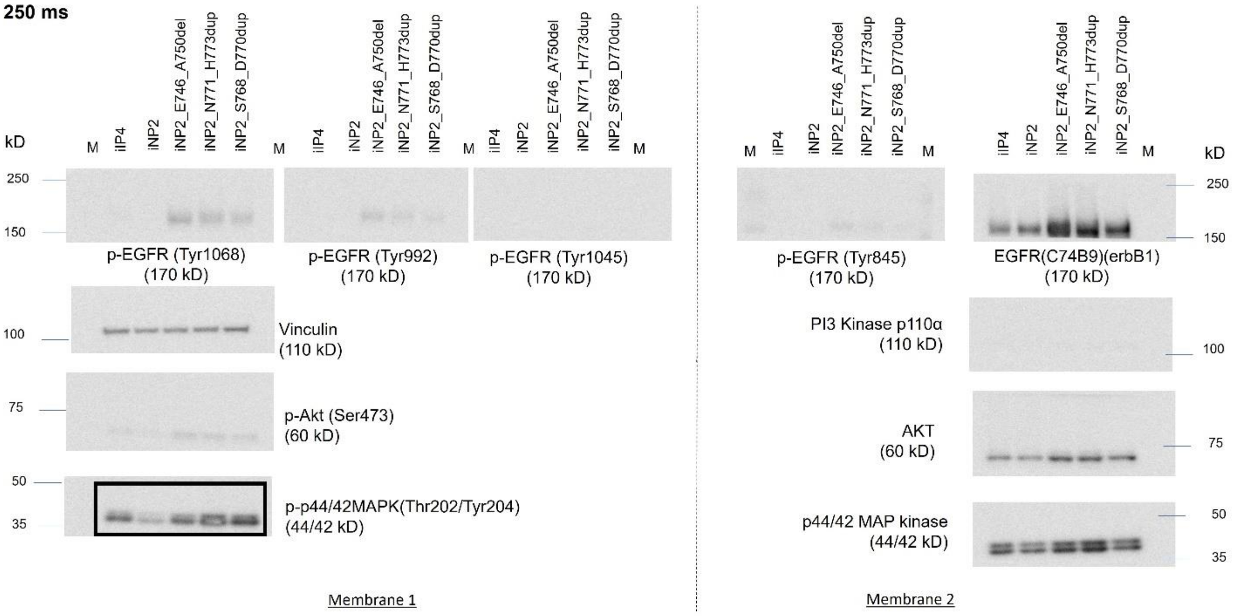

3 Sec

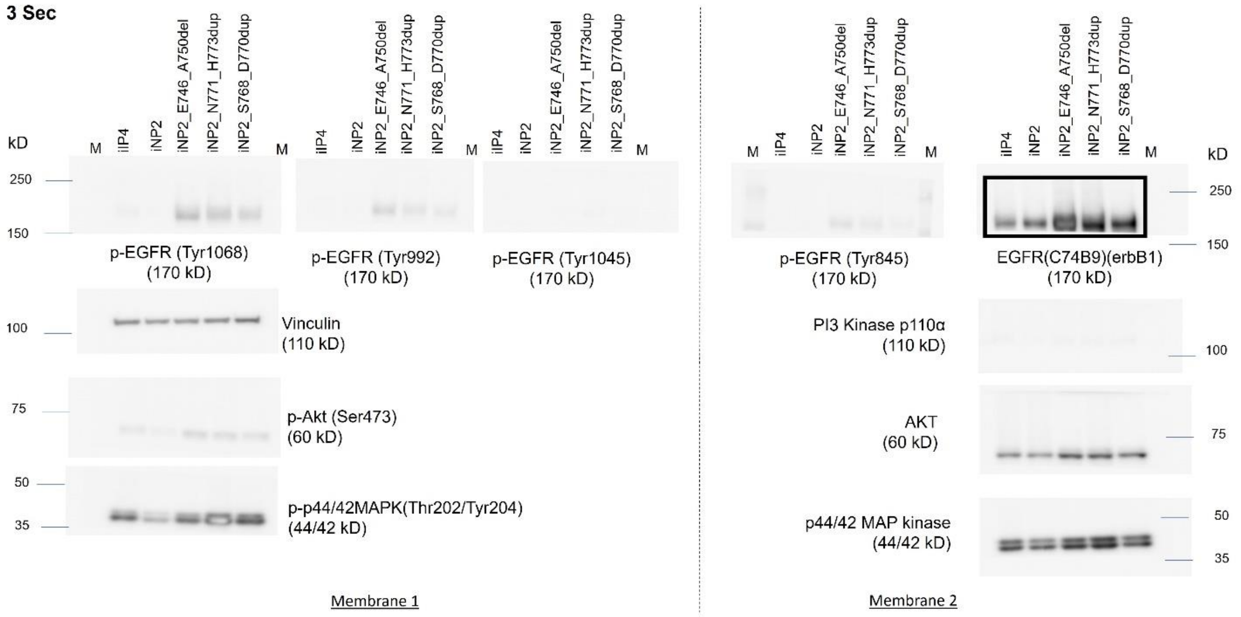

10 Sec

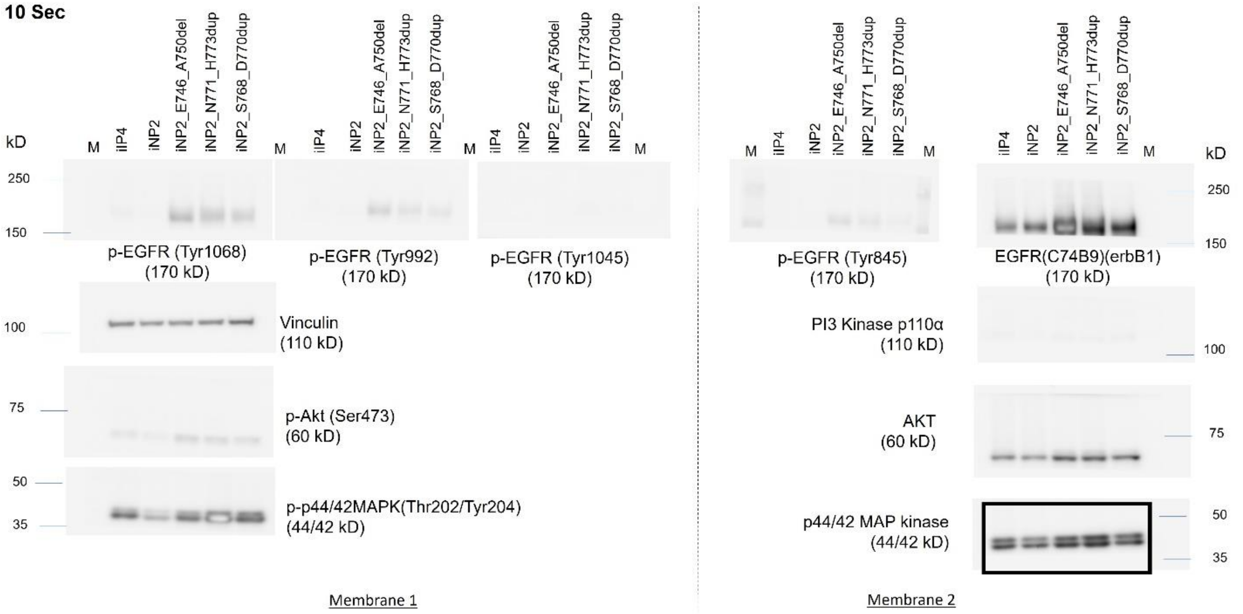

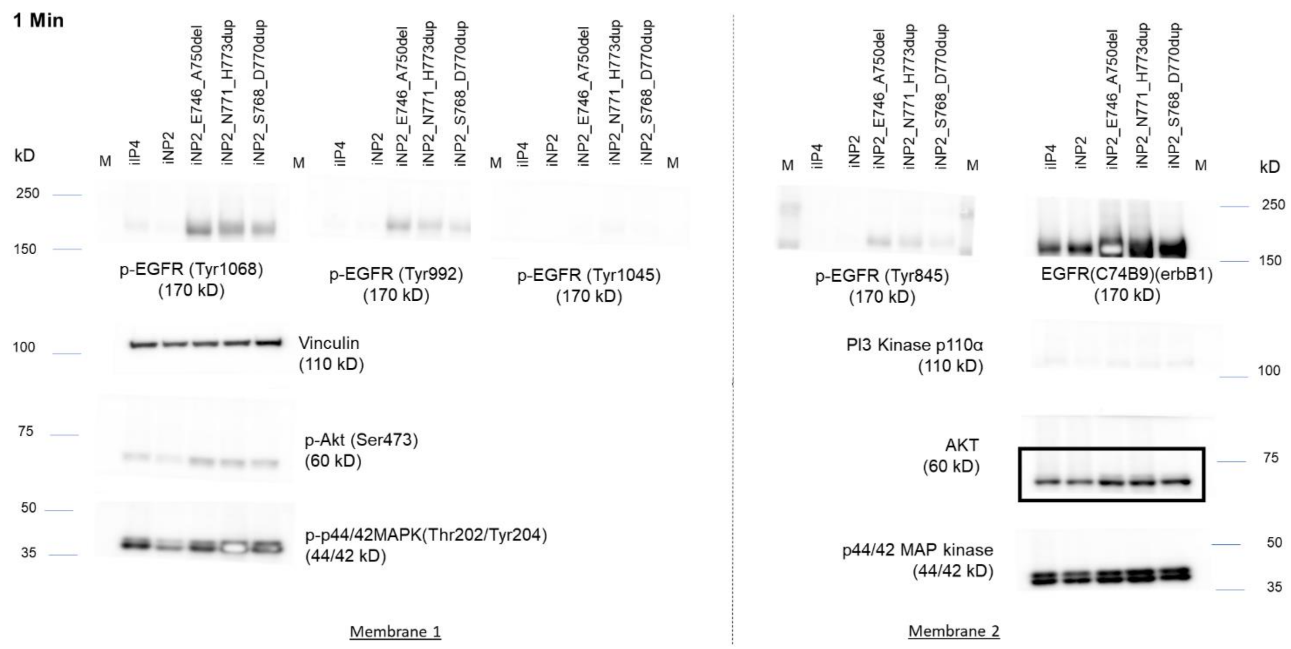
1 Min
kD
M
iIP4
iNP2
iNP2_E746_A750del
iNP2_N771_H773dup
iNP2_S768_D770dup
250
150
100
75
50
35
p-EGFR (Tyr1068) (170 kD)
p-EGFR (Tyr992) (170 kD)
p-EGFR (Tyr1045) (170 kD)
Vinculin (110 kD)
p-Akt (Ser473) (60 kD)
p-p44/42MAPK(Thr202/Tyr204) (44/42 kD)
Membrane 1
p-EGFR (Tyr845) (170 kD)
EGFR(C74B9)(erbB1) (170 kD)
PI3 Kinase p110α (110 kD)
AKT (60 kD)
p44/42 MAP kinase (44/42 kD)
Membrane 2

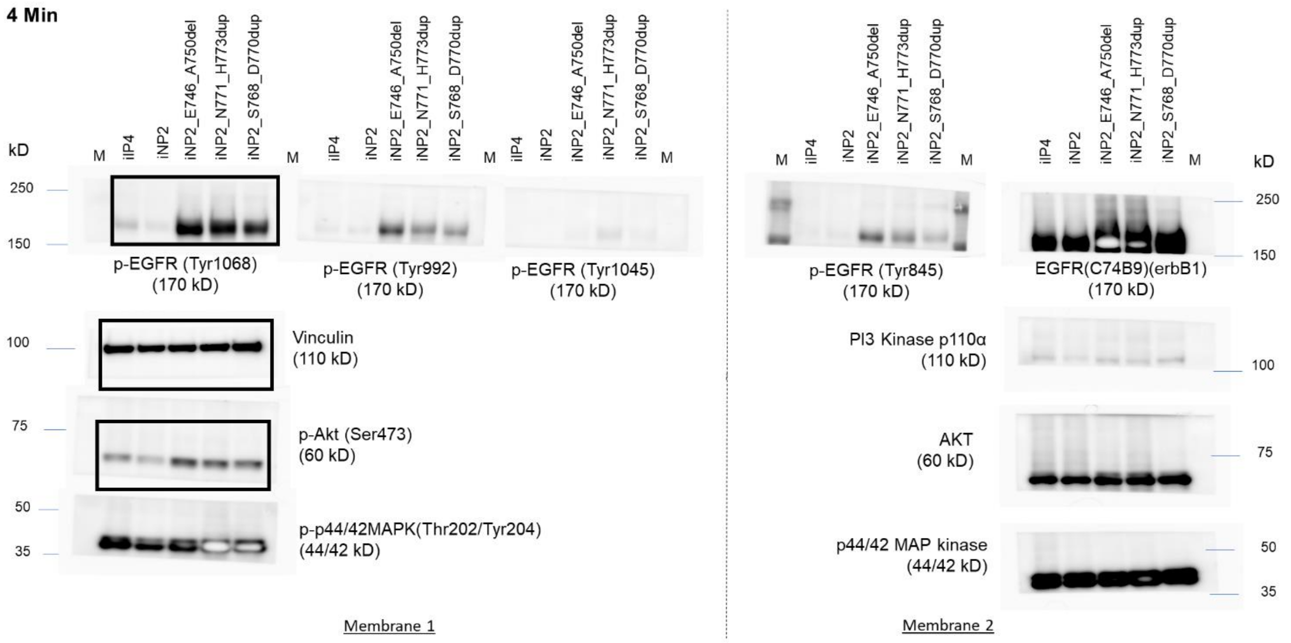
4 Min
kD
M
iIP4
iNP2
iNP2_E746_A750del
iNP2_N771_H773dup
iNP2_S768_D770dup
250
150
100
75
50
35
p-EGFR (Tyr1068) (170 kD)
p-EGFR (Tyr992) (170 kD)
p-EGFR (Tyr1045) (170 kD)
Vinculin (110 kD)
p-Akt (Ser473) (60 kD)
p-p44/42MAPK(Thr202/Tyr204) (44/42 kD)
Membrane 1
p-EGFR (Tyr845) (170 kD)
EGFR(C74B9)(erbB1) (170 kD)
PI3 Kinase p110α (110 kD)
AKT (60 kD)
p44/42 MAP kinase (44/42 kD)
Membrane 2

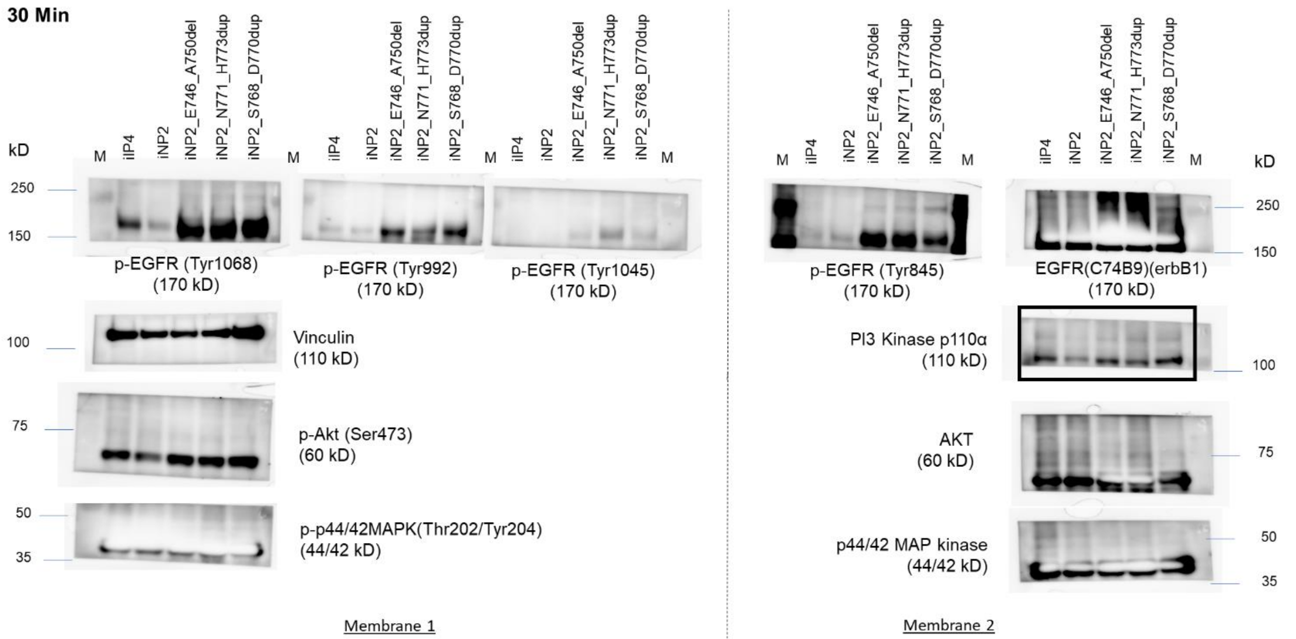
30 Min
kD
M
iIP4
iNP2
iNP2_E746_A750del
iNP2_N771_H773dup
iNP2_S768_D770dup
250
150
100
75
50
35
p-EGFR (Tyr1068) (170 kD)
p-EGFR (Tyr992) (170 kD)
p-EGFR (Tyr1045) (170 kD)
Vinculin (110 kD)
p-Akt (Ser473) (60 kD)
p-p44/42MAPK(Thr202/Tyr204) (44/42 kD)
Membrane 1
p-EGFR (Tyr845) (170 kD)
EGFR(C74B9)(erbB1) (170 kD)
PI3 Kinase p110α (110 kD)
AKT (60 kD)
p44/42 MAP kinase (44/42 kD)
Membrane 2

Cropped image

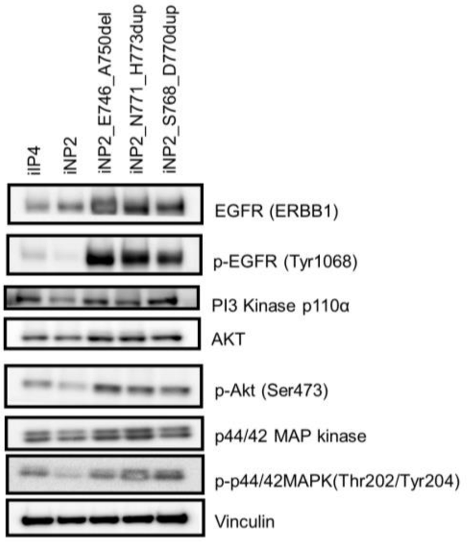

Figure S2. Full images of Western blot in Figure 3A. After the western blot was performed as described in Materials and Methods, to hybridize one blot with several antibodies at the same time, the membrane was cut into several pieces at desired positions prior to hybridization with specific antibodies. The figure shows the original images of western blot in different exposure times (250 milliseconds to 4 minutes in replicate 1, and 250 milliseconds to 30 minutes in replicate 2), the grouping of blots in Figure 3A was cropped from the same samples loaded in 2 gels with 8 blots. The black box in the original pictures of replicate 1 indicated group of the cropped blots that show in Figure 3A.

Replicate: 1

Original blots in different times (a time-lapse sequence)

30 Sec

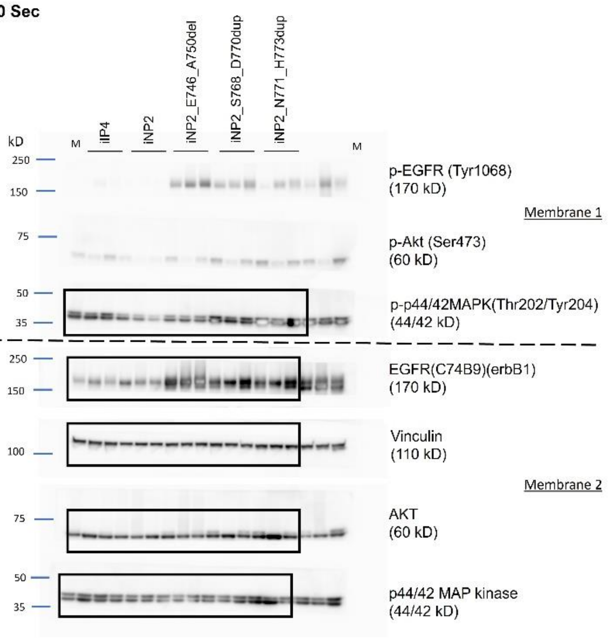

1 Min

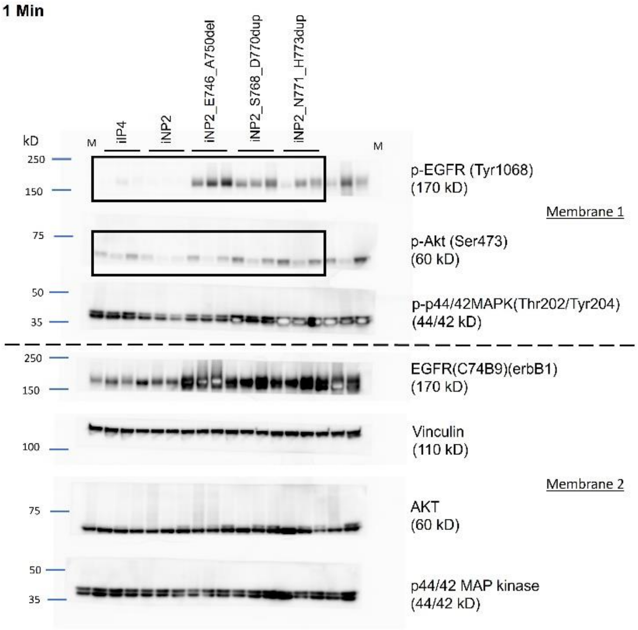

2 Min

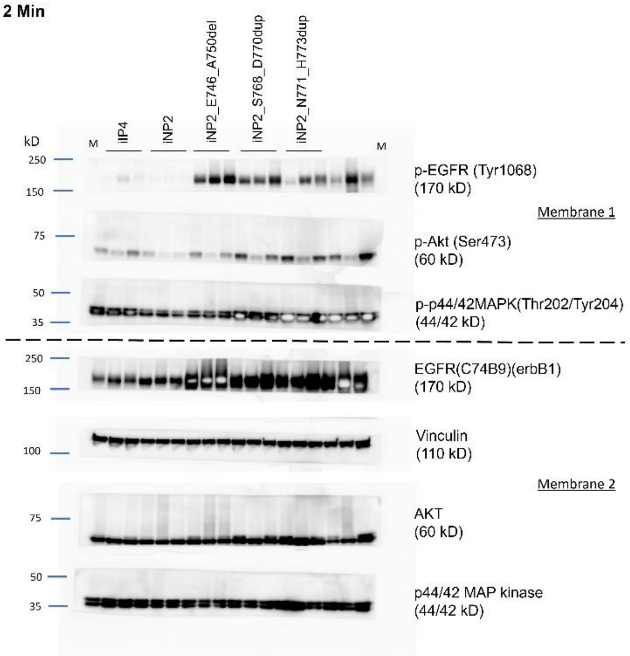

4 Min

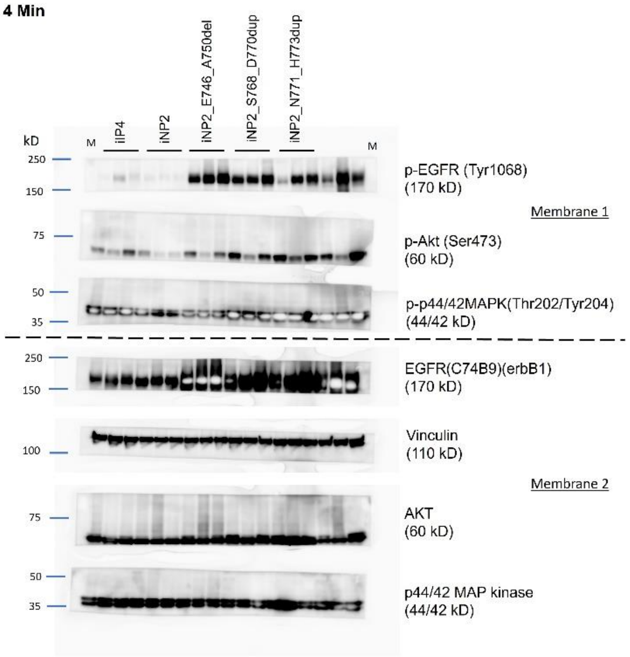

Cropped image in Figure 5A

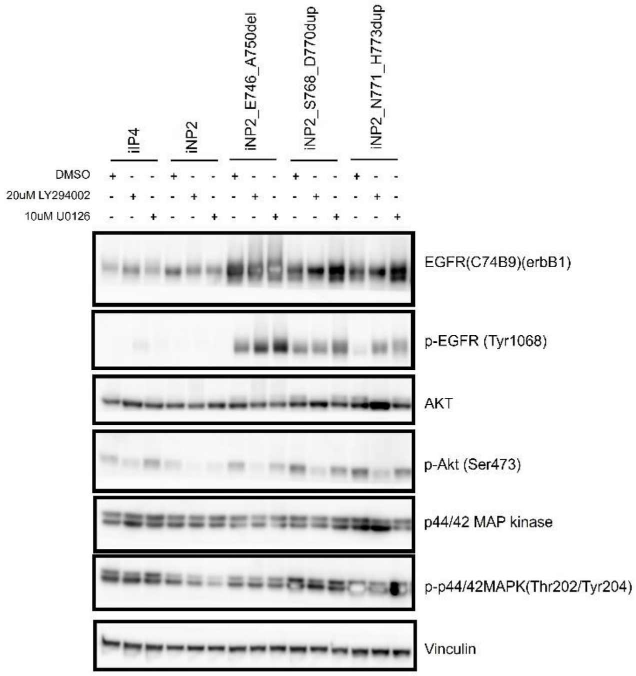

Replicate: 2 **Original blots in different times (a time-lapse sequence)**

15 Sec

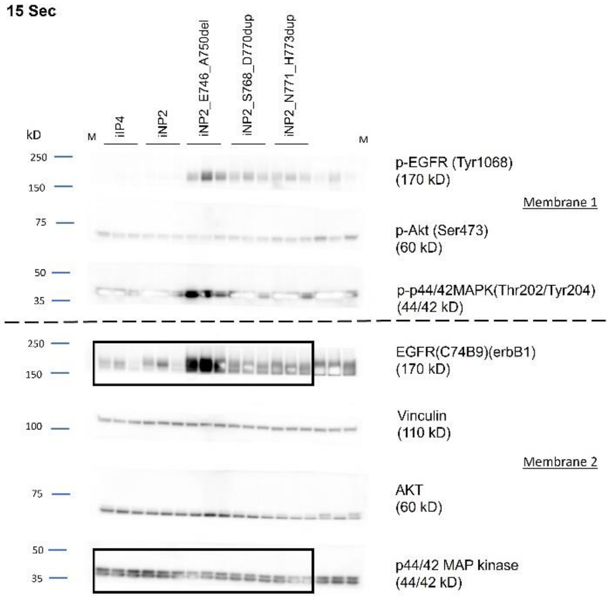

30 Sec

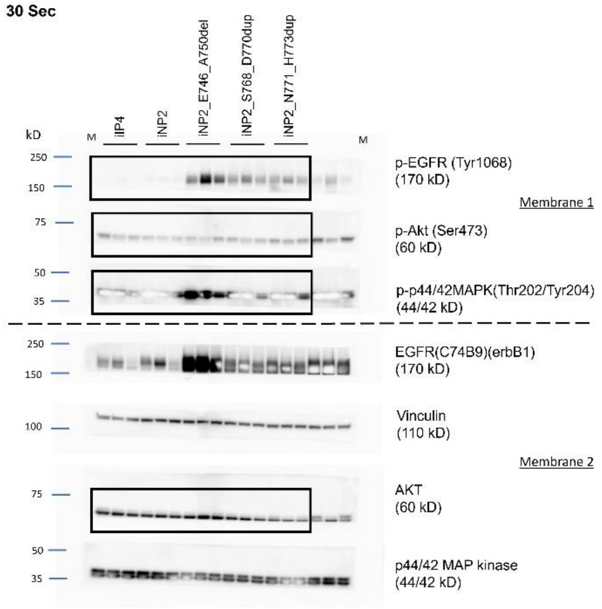

1 Min

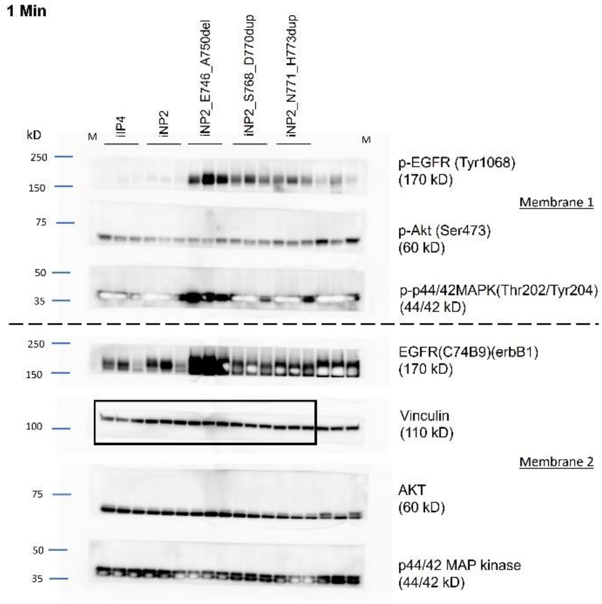

2 Min

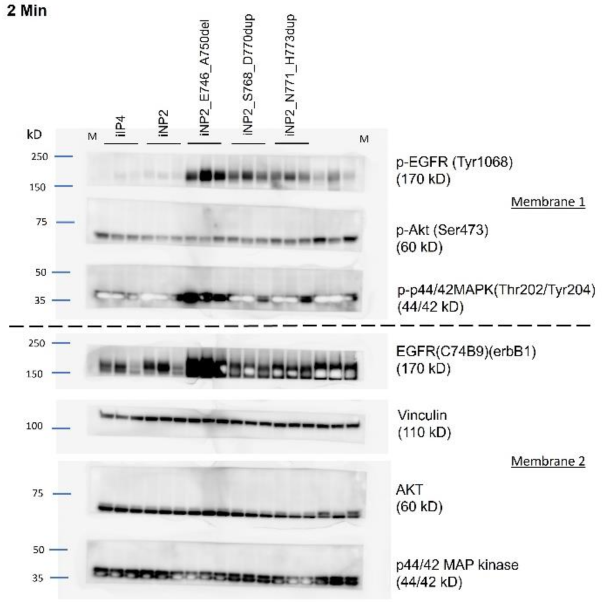

Cropped image

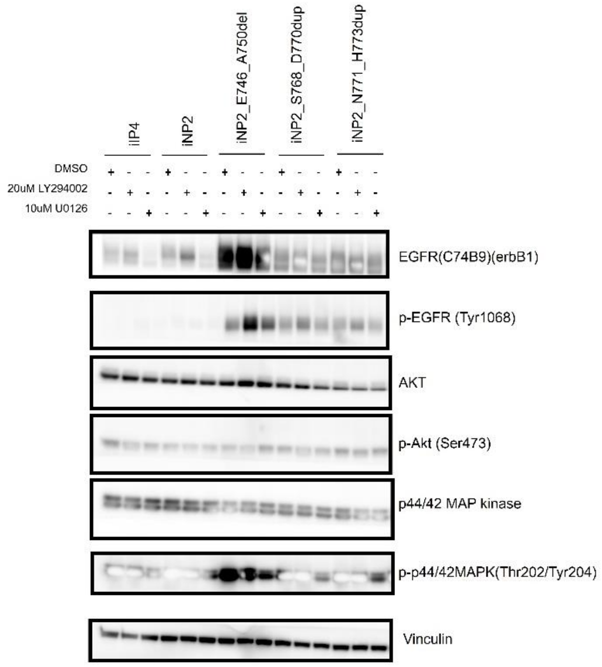

Figure S3. Full images of Western blot in Figure 5A. After the western blot was performed as described in Materials and Methods, to hybridize one blot with several antibodies at the same time, the membrane was cut into several pieces at desired positions prior to hybridization with specific antibodies. The figure shows the original images of western blot in different exposure times (30 seconds to 4 minutes in replicate 1, and 15 seconds to 2 minutes in replicate 2), the grouping of blots in Figure 5A was cropped from the same samples loaded in 2 gels with 7 blots. The black box in the original pictures of replicate 1 indicated a group of the cropped blots that show in Figure 5A.

Vector construction

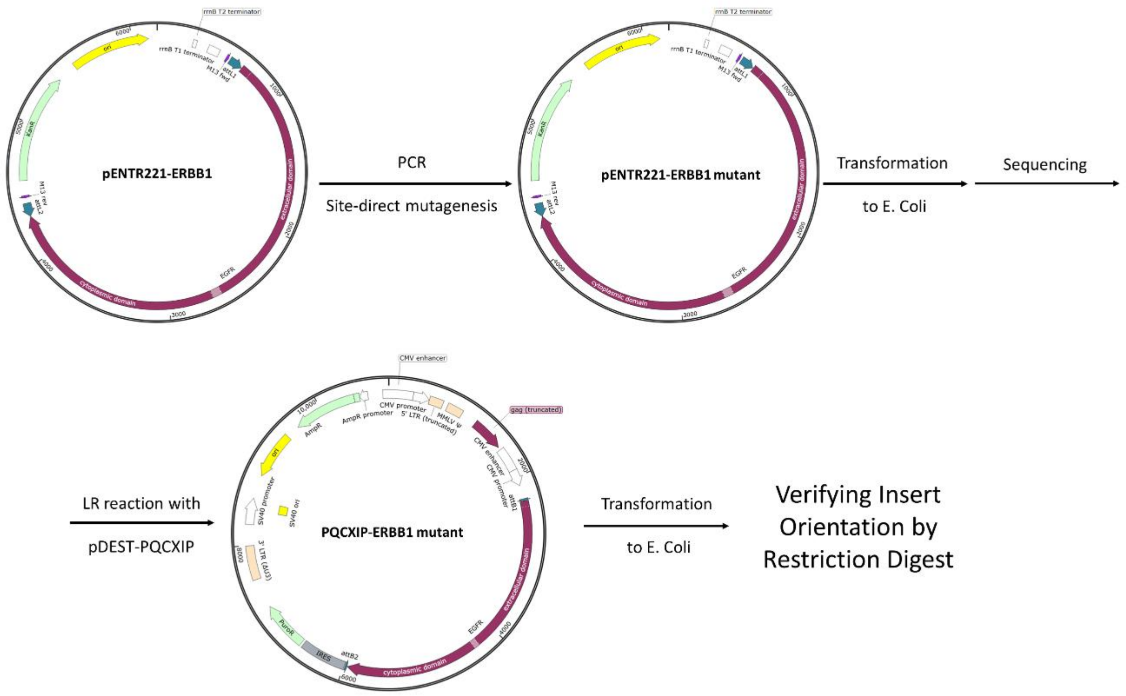

Figure S4. Protocol for ERBB1 mutant vector construction and verification.

Table S1: Primer sequences for site-directed mutagenesis and sequencing.

Target	Primer sequence
**Site-directed mutagenesis:**	
ERBB1_E746_A750del-F	5'-gtcgctatcaaggaaccaacatctccgaaa-3'
ERBB1_E746_A750del-R	5'-tttcggagatgttggttccttgatagcgac-3'
ERBB1_S768_D770dup-F	5'-gacAGCGTGGACaacccccacgtgtgccgcctgctg-3'
ERBB1_ S768_D770dup-R	5'-gttGTCCACGCTgtccacgctggccatcacgtaggcttc-3'
ERBB1_N771_H773dup-F	5'-cacAACCCCCACgtgtgccgcctgctgggcatctg-3'
ERBB1_N771_H773dup-R	5'-cacGTGGGGGTTgtgggggttgtccacgctggccatc-3'
**Sequencing:**	
ERBB1_2002F	5'-atgcgaaggcgccacatcgt-3'

pENTR221-ERBB1_S768_D770dup

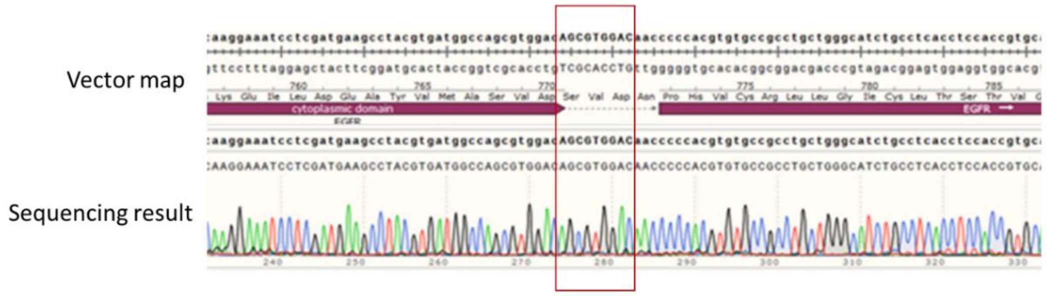

pENTR221-ERBB1_N771_H773dup

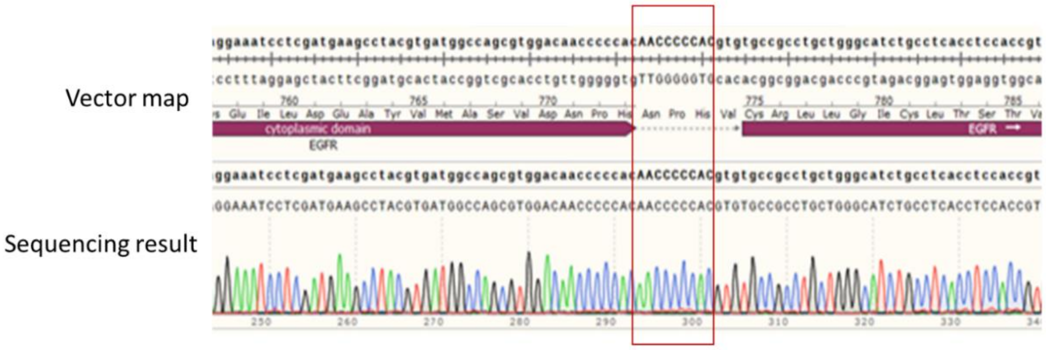

Figure S5. DNA sequencing result of the ERBB1 mutant vectors. After site-direct mutagenesis, the generated vectors were tranformed into E. Coli, clone was selected and extracted for the DNA. The DNA from each selected clones were used as template for DNA sequencing to obseved the specific mutation. Red box indicated insetion mutation at specific site in pENTR221-ERBB1_S768_D770dup and pENTR221-ERBB1_N771_H773dup vectors.

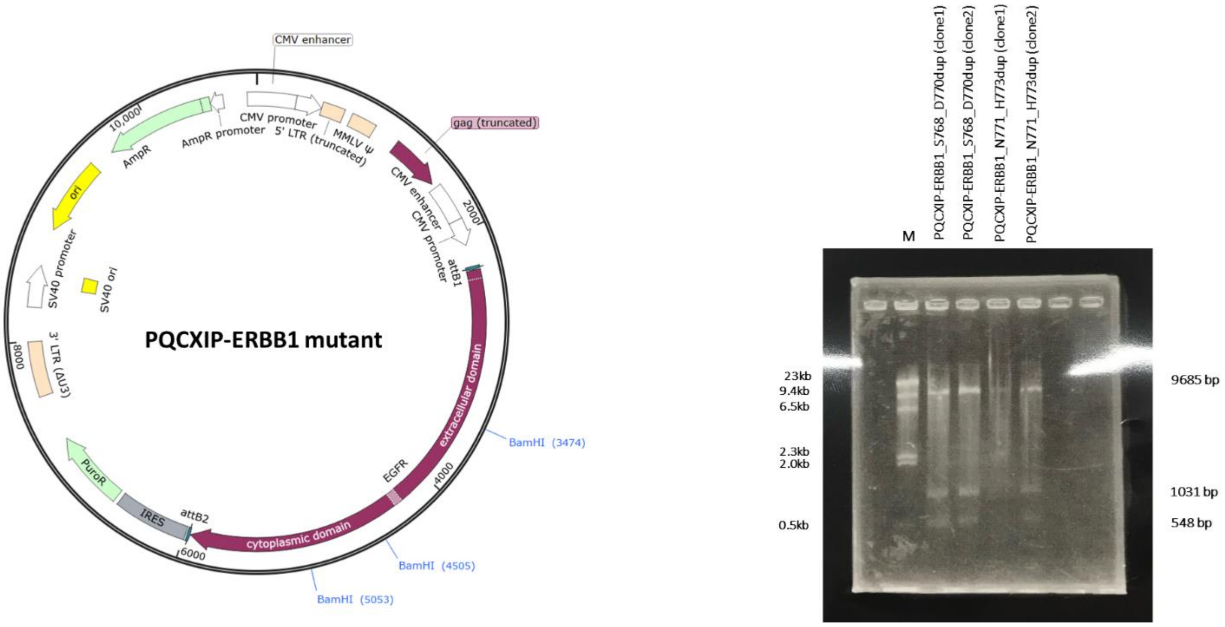

Figure S6. Verifying Insert Orientation by Restriction Digest. The restriction enzyme, BamH I was used to verify the ERBB1 gene which was inserted into PQCXIP vector. BamH I digested at specific site located in ERBB1 sequence and generated 3 fragments of digested ERBB1 gene.
